# Perceived stress and hair cortisol concentration in a study of Mexican and Icelandic women

**DOI:** 10.1371/journal.pgph.0000571

**Published:** 2022-08-03

**Authors:** Rebekka Lynch, Mario H. Flores-Torres, Gabriela Hinojosa, Thor Aspelund, Arna Hauksdóttir, Clemens Kirschbaum, Andres Catzin-Kuhlmann, Martín Lajous, Unnur Valdimarsdottir

**Affiliations:** 1 Centre of Public Health Sciences, University of Iceland, Reykjavik, Iceland; 2 Center for Research on Population Health, National Institute of Public Health, Mexico City, Mexico; 3 Department of Epidemiology, Harvard T. H. Chan School of Public Health, Boston, MA, United States of America; 4 Department of Psychology, TU Dresden, Dresden, Germany; 5 Department of Medicine, National Institute of Medical Sciences and Nutrition, Mexico City, Mexico; 6 Escuela de Medicina, Instituto Tecnológico y de Estudios Superiores de Monterrey, Monterrey, Mexico; 7 Department of Global Health and Population, Harvard T. H. Chan School of Public Health, Boston, MA, United States of America; 8 Department of Medical Epidemiology and Biostatistics, Karolinska Institutet, Solna, Sweden; University of East Anglia, UNITED KINGDOM

## Abstract

Hair cortisol concentration (HCC) represent a potential biomarker of chronic psychological stress. Previous studies exploring the association between perceived stress and HCC have been limited to relatively small and selected populations. We collected hair samples from 881 women from the Mexican Teachers’ Cohort (MTC) and 398 women from the Icelandic SAGA pilot-cohort following identical protocols. HCC was quantified using liquid chromatography coupled with tandem mass spectrometry. The self-reported Perceived Stress Scale (PSS, 10 and 4 item, range 0–40 and 0–16) was used to assess psychological stress. We conducted multivariable linear regression analyses to assess the association between perceived stress and log-transformed HCC in the combined sample and in each cohort separately. MTC participants had slightly higher HCC and PSS scores than SAGA participants (median HCC 6.0pg/mg vs. 4.7pg/mg and mean PSS-10 score 12.4 vs. 11.7, respectively). After adjusting for sociodemographic factors and health behaviors, we observed a 1.4% (95% CI 0.6, 2.1) increase in HCC for each unit increase in the PSS-10 score in the combined sample. Furthermore, PSS-10 quintiles were associated with a 24.3% (95% CI 8.4, 42.6, mean logHCC 1.8 vs 1.6) increase in HCC when comparing the highest to the lowest quintile, after multivariable adjustment. Similar results were obtained when we analyzed each cohort separately and when using the PSS-4. Despite relatively small absolute differences, an association between perceived stress and HCC was found in a sample of women from two diverse geographical and cultural backgrounds supporting the hypothesis that HCC is a viable biomarker in studies of chronic psychological stress.

## Introduction

Stress, despite its colloquial connotations, is a scientific term used to describe cognitive, emotional and physiologic reactions resulting from an imbalance between demands and resources as perceived by the individual [1]. This definition puts less weight on the nature of the event in question, rather it is the individual’s perception of the event that is paramount. Within this framework, the Perceived Stress Scale (PSS) was developed by Cohen et al [2,3] and has been widely used in research studies. Results from these studies provide evidence of the association of high PSS scores with psychiatric and somatic outcomes, including symptoms of depression and anxiety [4], increased susceptibility for infections [5] and cardiovascular disease [6]. However, the biological mechanisms of the stress-disease pathway in humans are poorly understood, partly due to the inherent complexities of measuring the stress response.

Cortisol is a hormone continuously secreted by the hypothalamic–pituitary–adrenal (HPA) axis in a diurnal rhythm but also in response to stress [7]. Cortisol levels can be measured in blood, urine, and saliva; however, these measurements may be more suited to measure acute stress responses, as their concentration changes rapidly, which may contribute to conflicting results in the stress literature [8]. Measuring cortisol concentration in hair has been proposed as an alternative way to consistently assess cumulative exposure to this stress-related hormone over a period of weeks to months [9], as well as a much simpler and non-invasive method of assessment. However, hair cortisol concentration differs both by gender and changes through the life course [10].

A 2017 meta–analysis of 2,441 individuals from 26 studies did not find an overall association between perceived stress and hair cortisol concentration (HCC) [10]. However, the study reported an increase in HCC in subpopulations with ongoing life conditions of severe stress, defined as exposure to a psychological condition that is threatening and/or exceeds coping resources that either persisted for at least one month or could be appraised as threatening for a month (e.g. trauma). The included studies were based on small samples (the majority with fewer than 100 participants) and very selected populations, e.g. fulltime dementia caregivers [11], soldiers [12]. Additionally, the majority of studies have been conducted in European populations, with only one study performed in Latin America [13], and used different assaying protocols to measure HCC. We aimed to assess the association between perceived stress and HCC in two culturally and geographically distinct samples of adult women from population-based studies in Mexico and Iceland. We hypothesized that HCC might be a viable biomarker for chronic psychological stress among women across these two different cultures.

## Materials and methods

### Study population

#### The Mexican Teachers’ Cohort (MTC)

The MTC is a prospective, closed cohort study of 115,314 women teachers, aged 25 years and older, that began in 2006–2008 in twelve geographically and economically diverse states in Mexico [14]. At baseline, and every three years, participants responded to questionnaires on their demographic and reproductive characteristics, lifestyle, and health. Between May 2016 and June 2017, a random sample of 2,003 study participants (aged ≥40 years and living within a 50 km radius from a clinical site) in two Mexican states were invited to take part in an ancillary study on stress and cardiovascular disease, among others. A clinical evaluation took place in two sites: the city of Monterrey, Nuevo Leon, and Mexico City. Close to 60% (n = 1,145) of those invited participated in the study.

#### The SAGA Cohort

The SAGA Cohort is a nationwide study on the impact of trauma on women’s health, that launched in 2018, with a recruitment period of one year [15]. The target population is all women, 18–69 years old, residing in Iceland. In the pilot study performed in 2014, a random sample of 689 adult women living in the greater Reykjavik area and attending the national Cancer Detection Clinic were invited to participate. All women had appointments for routine breast and cervical cancer screens from February through April of 2014. Nationally, 85% and 92% of eligible Icelandic women have attended the respective screens at least once [16]. Over 70% (n = 509) of the invited women responded to the online questionnaire and then attended the clinical visit in conjunction with their screening.

### Inclusion and exclusion criteria

For the present study, we included all women, 70 years or younger, who provided a hair sample at a clinical visit in both studies (1,111 from the MTC, and 492 from SAGA). Every woman from the MTC that attended a clinical visit donated a hair sample, but ten women from SAGA did not. We excluded pregnant women (3 (0.6%) from SAGA) (as cortisol levels rise during pregnancy [17]), women with an incomplete or incorrectly completed 10-item PSS-10 (79 (7.1%) from the MTC, and 33 (6.7%) from SAGA), and those with undetectable cortisol values (77 (6.9%) from the MTC and 23 (4.7%) from SAGA). We also excluded women with a cortisol: cortisone ratio > 3 (74 (6.7%) from the MTC and 35 (7.1%) from SAGA), as these values are implausible and probably due to exogenous cortisol [18,19]. Thus, the final study sample comprised 1,279 women, 881 from the MTC and 398 from SAGA.

All women provided written informed consent. In Mexico, the institutional review boards at the National Institute of Public Health and the School of Medicine and Health Sciences of the Monterrey Institute of Technology approved the project. In Iceland, the study was approved by the National Bioethics Committee (VSNb2013010025/03.07) and reported to the Icelandic Data Protection Authority. Data transfer was approved by the Mexican institutional review boards and combined analyses were performed at the University of Iceland.

### Assessment of hair cortisol concentration (HCC)

For participants in both countries (Mexico and Iceland), hair samples were collected during the clinical visit to measure cortisol and cortisone levels. Samples were taken from the posterior vertex by study personnel, if there was at least 3cm of hair growth, and stored in aluminum foil. As hair grows an estimated 1 cm per month, the 3 cm closest to the scalp represent the prior three months [20]. The hair samples were stored at room temperature in a dark and dry environment. The samples were centrally assayed to determine hair cortisol and cortisone levels at the Dresden University of Technology (TU Dresden) in Germany under the supervision of one of the coauthors (CK). Liquid chromatography coupled with tandem mass spectrometry (LCMS/MS) was used to measure cortisol and cortisone values in the hair samples (see Gao et al [21] for a more detailed description).

### Assessment of perceived stress

Stress was assessed with the 10-item PSS-10, developed by Cohen et al [3]. The PSS-10 focuses on “the degree to which respondents found their lives unpredictable, uncontrollable, and overloading” [3], rather than assessing a specific life-event. The scale covers the past month, using a 5-point response scale (0 = “never”, 1 = “almost never”, 2 = “sometimes”, 3 = “fairly often”, 4 = “very often”). As per standard practice PSS-10 scores were obtained by reversing the scores on the four positive items (4, 5, 7 and 8) and summing across all 10 items, with higher scores indicating higher levels of perceived stress (scores range from 0 to 40) [22]. We additionally calculated a shorter PSS-4 score based on questions 2, 4 (reversed), 5 (reversed) and 10 of the original PSS-10 (scores range from 0–16), as its brevity could make it valuable for larger epidemiological studies. Since the PSS is not a diagnostic instrument and does not have established cutoff values, we categorized participants’ scores using quintiles of the PSS-10 total scores. Participants responded to a paper questionnaire that included the PSS-10 during the clinical visit for the Mexican sample in Spanish and in an online questionnaire for the Icelandic sample in Icelandic. Internal consistency for the full scoring scale was previously found to be high (Cronbach’s alpha r = 0.83) in a validation study of 1,310 MTC participants [23] and is similar in a post-hoc analysis of the current Icelandic sample (r = 0.82).

### Covariate assessment

Other variables were selected and harmonized to compare characteristics of participants from both countries based on availability and proposed frameworks for studying stress in human populations [24]. Missing values were imputed to the most frequent value (categorical) or the median value (numerical) for each variable, with 89.1% of MTC participants and 96.7% of SAGA participants having complete data. S1 Table includes detailed information on variable selection, harmonization and missingness. The sociodemographic factors assessed in both samples were age, marital status, education level and employment status. Information on health behaviors including alcohol consumption, smoking status and body mass index (measured clinically) was also collected in both cohorts.

### Statistical analysis

We combined data from both cohort studies to maximize power and assess general patterns in the distribution of the effects. We explored the distribution of participant characteristics and the graphical distribution of PSS scores and HCC in the combined sample and in each cohort separately. HCC was log-transformed to normalize its distribution. We fit linear regression models adjusted by age and cohort (only for the overall sample) to estimate the percentage difference in mean HCC and 95% confidence interval (95% CI). We repeated the analyses using the quintiles of PSS-10 and -4 instead of a continuous PSS score, with the lowest quintile as the reference. To test for trend, we assigned the median PSS score within each quintile and fit a model with the exposure as a continuous variable. Finally, we re-ran analyses as a multivariable model with age, cohort (only for the overall sample), marital status, occupation, educational level, BMI (continuous), and smoking status, as these present possible confounders in the association between perceived stress and HCC. Analyses were conducted using R version 3.4.2.

## Results

Characteristics of study participants in the combined sample and by cohort (881 from MTC and 398 from SAGA) are presented in Table 1. In the combined sample, the mean age was 51.2 (SD 7.7), with the majority married (71.2%) and employed (89.8%). The mean BMI for participants was 28.9 (5.8), with 12.9% current smokers. MTC participants were on average younger and less likely to be ≥60 years than SAGA participants. MTC were also more likely to be obese, and less likely to smoke or consume alcohol compared with SAGA participants. When we explored the distribution of the combined sample’s participant characteristics by quintiles of PSS-10 scores (S2 Table), we observed that, compared with women in the lowest quintile, those in the highest quintiles were younger, less likely to have a graduate degree or to be married, as well as more likely to report other occupation status. Women in the highest quintiles were also more likely to be obese and to currently smoke.

**Table 1:** Background characteristics of participants in the Mexican Teacher’s Cohort (MTC) and the Icelandic SAGA Cohort, via questionnaire data, by cohort and after pooling the data.

	Overall N = 1279	MTC N = 881	SAGA N = 398
**Hair Cortisol Concentration**			
Median cortisol (pg/mg)	5.5	6.0	4.7
25^th^ -75^th^ percentile	3.4–8.9	3.7–9.5	3.0–7.6
Mean log-cortisol (SD)a	1.7 (0.8)	1.8 (0.8)	1.6 (0.7)
**Mean age (SD)a**	51.2 (7.7)	50.9 (6.0)	52.0 (10.6)
**Age in years (%)**			
20–39	72 (5.6)	40 (4.5)	32 (8.0)
40–49	386 (30.2)	268 (30.5)	118 (29.6)
50–59	664 (51.9)	522 (59.3)	142 (35.7)
60–70	157 (12.3)	51 (5.8)	106 (26.6)
**Graduate Degree (%)**	261 (20.4)	190 (21.6)	71 (17.8)
**Occupation (%)**			
Employed	1148 (89.8)	836 (94.9)	312(78.4)
Retired	62 (4.8)	45 (5.1)	17 (4.3)
Otherb	69 (5.4)	0 (0.0)	69 (17.3)
**Marriage/Cohabitation (%)**	911 (71.2)	619 (70.3)	292 (73.4)
**BMI**^**c**^ **(SD)**^**a**^	28.9 (5.8)	29.5 (5.8)	27.5 (5.6)
**BMI**^**c**^ **category (%)**			
Normal weight	328 (25.6)	182 (20.7)	146 (36.7)
Overweight	510 (39.9)	370 (42.0)	140 (35.2)
Obese	441 (34.5)	329 (37.3)	112 (28.1)
**Smoking (%)**			
Never	763 (59.7)	592 (67.2)	171 (43.0)
Former	351 (27.4)	177 (20.1)	174 (43.7)
Current	165 (12.9)	112 (12.7)	53 (13.3)
**Alcohol consumption (drinks/month) (SD)** ^**a**^	3.2 (5.0)	1.9 (3.9)	6.0 (5.8)
**Mean PSS** ^**d**^ **-10 score (SD)** ^**a**^	12.2 (6.0)	12.4 (6.0)	11.7 (6.0)
**Mean PSS** ^**d**^ **-4 score (SD)** ^**a**^	4.0 (2.7)	3.9 (2.7)	4.4 (2.8)

^a^ Standard deviation.

^b^ Unemployed, disability, homemaker or sick leave.

^c^ Body Mass Index.

^d^ Perceived Stress Scale.

The 1,279 study participants had a mean PSS-10 score of 12.2 (SD 6.0) (Table 1). The mean HCC before log transformation was 8.1 pg/mg (SD 19.1) and the median was 5.5 pg/mg (IQR 3.4–8.9 pg/mg) (S1 Fig, panel A). After log transformation, mean HCC was 1.7 (0.8) and the median was 1.7 (IQR 1.2–2.2) (S1 Fig, panel B). MTC participants had, on average, both slightly higher PSS-10 scores and higher log-transformed HCC than SAGA participants (12.4 vs 11.7 and 1.8 vs 1.6, respectively) (Table 1 and S2 Fig). The same pattern was not observed for the shorter PSS-4, with a mean score of 4.0 (SD 2.7), and higher values of PSS-4 among SAGA participants (4.4 vs 3.9) (Table 1).

**Fig 1:**
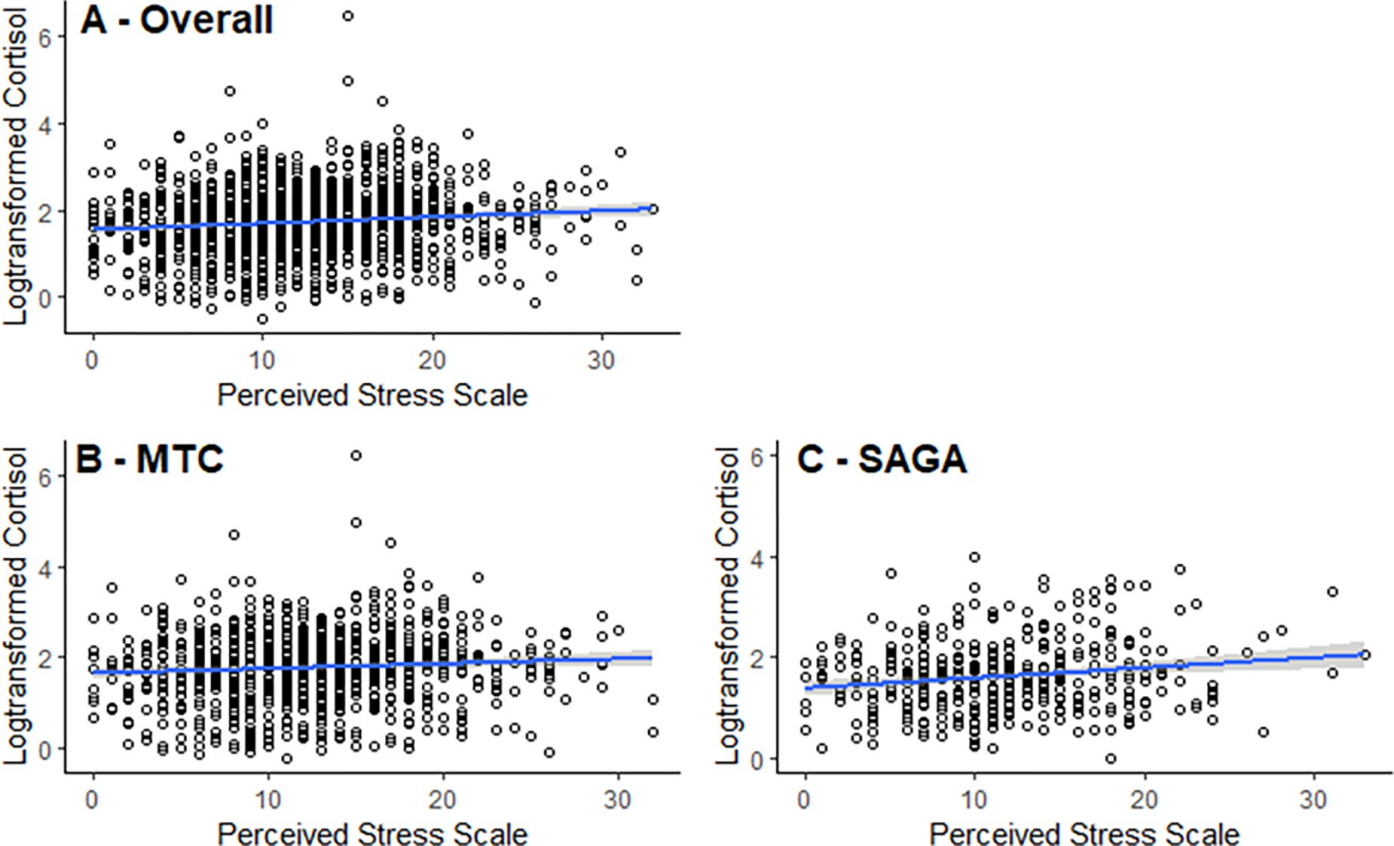
Multivariable adjusted linear regression for log-transformed cortisol with 95% confidence intervals, with data from the Mexican Teacher’s Cohort (MTC) (N = 881) and the Icelandic SAGA Cohort (N = 398), both as a combined sample and by cohort according to 10-item Perceived Stress Scale score. The figures are scatterplots with an overlying linear regression and 95% confidence intervals. The results are adjusted for marital status, occupation, educational level, BMI, smoking status as well as cohort for the combined sample. The overall sample (Panel A) had a 1.4% (95% CI 0.6, 2.11) increase in log-cortisol per unit increase of PSS. The Mexican sample (Panel B) had a 1.3% (95% CI –0.7, 3.3) increase in log-cortisol. The Icelandic sample (Panel C) had a 2.0% (95% CI 0.7, 3.2) increase in log-cortisol. See S5 Fig for age-adjusted results.

We observed a linear association between perceived stress (based on the PSS-10 score) and HCC (Fig 1, panel A). After multivariable adjustment, we found that a unit increase in the PSS-10 score was associated with 1.4% (95% CI 0.6, 2.1) increase in mean HCC. Results by country demonstrated an increase of 1.3% (95% CI -0.7, 3.3) in mean HCC by unit increase in the PSS-10 score in the MTC and 2.0% (95% CI 0.7, 3.2) in the SAGA. We observed similar results in age-adjusted models (S3 Fig). Likewise, the shorter PSS-4 results indicate a 2.3% (95% CI 0.7, 4.0) increase in HCC for each unit increase in the PSS-4 score in the combined sample after adjusting for age. Estimates varied somewhat when each country sample was analyzed independently, with a non-significant increase (1.4%, 95% CI -0.6, 3.4) in the MTC sample and a significant increase of 4.3% (95% CI 1.6, 7.1) in the SAGA sample (S5 Fig, panels B and C).

**Fig 2:**
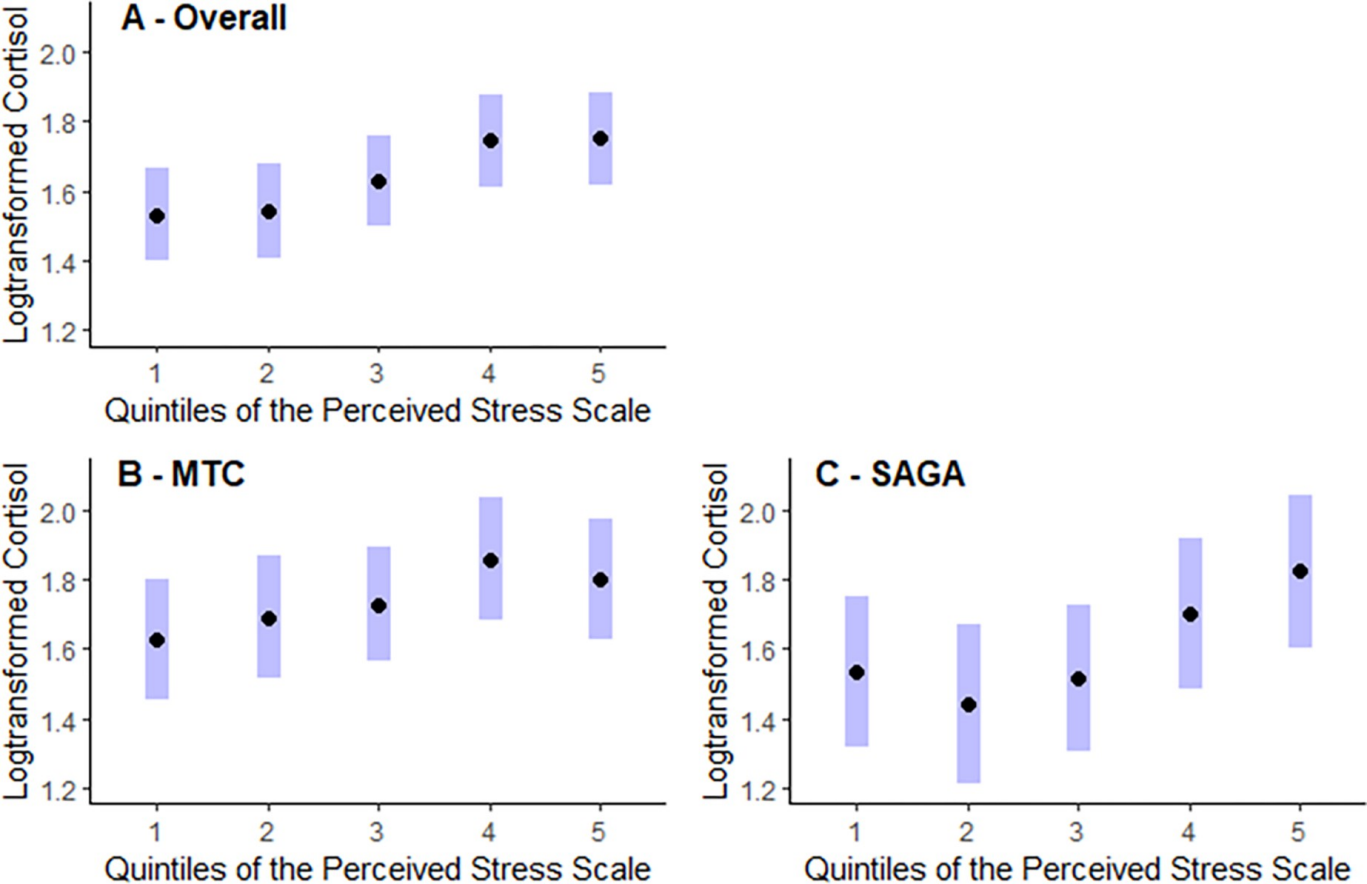
Differences in mean log-transformed cortisol by quintiles of the 10-item Perceived Stress Scale with data from the Mexican Teacher’s Cohort (MTC) (N = 881) and the Icelandic SAGA Cohort (N = 398), both as a combined sample and by cohort, with multivariable adjustment. The panels depict the mean log-cortisol value with the black dot representing the mean value and the bands representing 95% confidence intervals (logHCC with 95%CI) for each quintile of the Perceived Stress Scale (PSS). The results are adjusted for marital status, occupation, educational level, BMI, smoking status as well as cohort for the combined sample. Panel A includes the overall sample, panel B, the Mexican sample, and panel C the Icelandic sample. See S6 Fig for age-adjusted results.

Similar results were obtained when we explored perceived stress by quintiles of PSS-10 (Fig 2, panel A), with both raw and log-transformed cortisol levels increasing with each quintile of perceived stress (S2 Table). Women in the 5^th^ quintile had 24.3% (95% CI 8.4, 42.6) higher mean HCC after multivariable adjustment, compared with women in the lowest quintile (p-trend <0.001, mean logHCC 1.6 vs 1.8), and 24.1% (95% CI 8.3, 42.2) higher mean HCC after age adjustment. The data also indicates a plateau between the 4^th^ and 5^th^ quintile. A similar pattern was observed in both cohorts after age adjustment (S4 Fig, panels B and C), though the difference in HCC levels between the highest and lowest quintile of perceived stress appeared to be larger in SAGA (33.3%, 95% CI 5.7, 68.0, multivariable adjusted) and there was a decrease in the magnitude of the estimated effect from the 4^th^ to the 5^th^ quintile for the MTC (4^th^ quintile 26.0%, 95% CI 6.6, 48.9 and 5^th^ quintile 18.6%, 95% CI 0.2, 40.3). With regard to the PSS-4, the results were non-significant, but were suggestive of a J curve (S6 Fig).

## Discussion

In this study of two cohorts of women from the general population of Mexico and Iceland, we found a dose-response association between perceived stress and HCC. Women in the highest quintile of the Perceived Stress Scale had significantly higher HCC compared with the lowest quintile and this association remained unchanged after multivariable adjustment, though the absolute difference was small (mean logHCC 1.8 vs 1.6). This stepwise increase is also suggested when each cohort was examined separately, though a plateau was seen in the highest quintiles in the Mexican MTC sample.

To date, this is the largest study on perceived stress and HCC. One of its strengths is the harmonized assessments and centralized assaying of women from two considerably diverse ethnic and geographic backgrounds yielding largely similar results. Another strength of this study is that both cohorts were made up of women, as earlier research has shown significant differences in both hair cortisol concentration [10] and perceived stress [25] by gender. While the study is cross-sectional, the design fits nicely with the research question on the association between two dynamic measures of stress–self-reported and biological—at a single point in time. Disentangling causal direction is outside the scope of this study, however, our results are partly supported by previous studies examining hair cortisol repeatedly (two and four times) through a year at college [26] and a medical internship [27]. Both found that HCC increased in response to stressful events but did not find an association with the Perceived Stress Scale.

With two distinct cohorts, one an occupational cohort and the other a general cohort of cancer screening participants, and on two different continents, it is likely that the stressors faced are quite diverse. Nevertheless, although some differences were observed between cohorts regarding stress levels, both in terms of self-perceived stress and hair cortisol concentration, and absolute differences were small, this study demonstrates a positive association between self-perceived stress and hair cortisol levels across two cultures with diverse sources of stress.

A 2017 meta-analysis did not find an overall association between perceived stress and HCC [10]. The meta-analysis summarized the findings of mostly small-scale studies using varying measures of perceived stress which may contribute to the varying results. Two of the largest studies in this review did not use the Perceived Stress Scale but the 57-item Trier Inventory for the Assessment of Chronic Stress or its screening scale. Neither study found an association with hair cortisol concentration, one with 109 individuals from a convenience sample [28] and the other involving 654 older adults of both genders [29]. It is therefore possible that the Trier assessment does not adequately capture the facets of stress that are linked to cortisol dysregulation. Interestingly, a study of 37 couples (N = 74) that measured the Weekly Hassle Scale (WHS), the Perceived Stress Scale and the Triers Inventory for Assessment of Chronic Stress repeatedly over 12 weeks (12, 3, and 1 time respectively), and then assayed hair for cortisol found WHS alone predicted hair cortisol concentration. When using state-space modelling they found that all three explained an incremental variance portion of hair cortisol concentration–highlighting that no one scale perfectly captures hair cortisol changes [30].

Our results are more in line with a study of 324 Canadians, with oversampling of individuals with mental health problems or in abusive relationships, where a marginally positive association (Beta 0.107, unadjusted p = 0.057) was noted between the Perceived Stress Scale and hair cortisol concentration [31]. The curvilinear association reported in that study was similar to the results in the MTC cohort where the fourth quintile had the highest mean cortisol level.

The 2017 meta-analysis did find an association with on-going chronic stress, as defined by Miller et al [32], with a 43% increase in hair cortisol concentration. Of eight studies, half included some measure of PSS, with no study showing an association between hair cortisol levels and perceived stress, but all showing an association with event-based chronic stress. The largest of these studies included 85 caregivers [13], underscoring the lack of power that plagues earlier studies.

The PSS-4 is a shorter version of the Perceived Stress Scale and may be better suited for large epidemiological studies. Few studies have used this scale, though they exist [26,33]. Our main result, a positive linear association, remained the same when this smaller scale was used, however when divided into quintiles the association was slightly attenuated compared with the full PSS-10. This may reflect the fact that a smaller scale, although a valid tool, needs greater power to ascertain an association.

In summary, we found a modest positive association between perceived stress and hair cortisol concentration in two distinct cohorts of women, from Mexico and Iceland. The findings from earlier studies–using varying measures of stress—have been quite conflicting, however our findings indicate that the Perceived Stress Scale may in part reflect the underlying cortisol concentration in the general population of women. Further research is needed to assess this association, both regarding possible gender differences as well as through longitudinal study designs for further understanding of the temporal pattern in the association between perceived stress and HCC.

## Supporting information

S1 Fig Distribution of hair cortisol concentration in the Mexican Teacher’s Cohort (MTC) (N = 881) and the Icelandic SAGA Cohort (N = 398), before and after log-transformation. (TIFF)

S2 Fig Distribution of Perceived Stress Scale scores in the Mexican Teacher’s Cohort (MTC) (N = 881) and the Icelandic SAGA Cohort (N = 398). (TIFF)

S3 Fig Age-adjusted linear regression for log-transformed cortisol with 95% confidence intervals, with data from the Mexican Teacher’s Cohort (MTC) (N = 881) and the Icelandic SAGA Cohort (N = 398), both as a combined sample and by cohort according to 10-item Perceived Stress Scale score. The figures are scatterplots with an overlying linear regression and 95% confidence intervals. The overall sample (Panel A) is age and cohort adjusted, with an 1.3% (95% CI 0.6, 2.1) increase in log-cortisol per unit increase of PSS (p value 0.001). The Mexican sample (Panel B) is age adjusted, with a 1.0% (95% CI 0.1, 1.9) increase in log-cortisol (p value 0.04). The Icelandic sample (Panel C) is age adjusted, with a 2.1% (95% CI 0.9, 3.4) increase in log-cortisol (p value 0.004).
(TIF)

S4 Fig Differences in mean log-transformed cortisol by quintiles of the 10-item Perceived Stress Scale with data from the Mexican Teacher’s Cohort (MTC) (N = 881) and the Icelandic SAGA Cohort (N = 398), both as a combined sample and by cohort, adjusted for age and cohort (for the combined sample). The panels depict the mean log-cortisol value with the black dot representing the mean value and the bands representing 95% confidence intervals (logHCC with 95%CI) for each quintile of the Perceived Stress Scale (PSS). Panel A includes the combined sample, panel B the Mexican sample, and panel C the Icelandic sample. The overall sample is adjusted for age and cohort, and the country samples are adjusted for age only.
(TIF)

S5 Fig Age-adjusted linear regression for log-transformed cortisol with 95% confidence intervals, with data from the Mexican Teacher’s Cohort (MTC) (N = 881) and the Icelandic SAGA Cohort (N = 398), both as a combined sample and by cohort according to the 4-item Perceived Stress Scale score. The figures are scatterplots with an overlying linear regression and 95% confidence intervals. The overall sample (Panel A) is age and cohort adjusted, with a 2.3% (95% CI 0.7, 4.0) increase in log-cortisol per unit increase of the 4-item PSS (p value 0.010). The Mexican sample (Panel B) is age adjusted, with an 1.4% (95% CI -0.6, 3.4) increase in log-cortisol (p value 0.212). The Icelandic sample (Panel C) is age adjusted, with a 3.9% (95% CI 1.1, 6.7) increase in log-cortisol (p value 0.008).
(TIF)

S6 Fig Differences in mean log-transformed cortisol by quintiles of the 4-item Perceived Stress Scale with data from the Mexican Teacher’s Cohort (MTC) (N = 881) and the Icelandic SAGA Cohort (N = 398), both as a combined sample and by cohort, adjusted for age and cohort (overall sample). The panels depict the mean log-cortisol value with 95% confidence intervals (logHCC with 95%CI) for each quintile of the 4-item Perceived Stress Scale (PSS). Panel A includes the overall sample, panel B, the Mexican sample, and panel C the Icelandic sample. The overall sample is adjusted for age and cohort, and the country samples are adjusted for age.
(TIF)

S1 Table Description of variables used from the Mexican Teacher’s Cohort (MTC) (N = 881) and the Icelandic SAGA Cohort (N = 398), their harmonization and missingness. (DOCX)

S2 Table Characteristics of study participants (N = 1,279), combining data from the Mexican Teacher’s Cohort (MTC) and the Icelandic SAGA Cohort, by quintiles of the Perceived Stress Scale. (DOCX)
