# Supplementary figures and images for: Perceived stress and hair cortisol concentration in a study of Mexican and Icelandic women
Source: PLOS Glob Public Health. 2022 Aug 3;2(8):e0000571. doi: 10.1371/journal.pgph.0000571 (PMC10021558; doi:10.1371/journal.pgph.0000571)

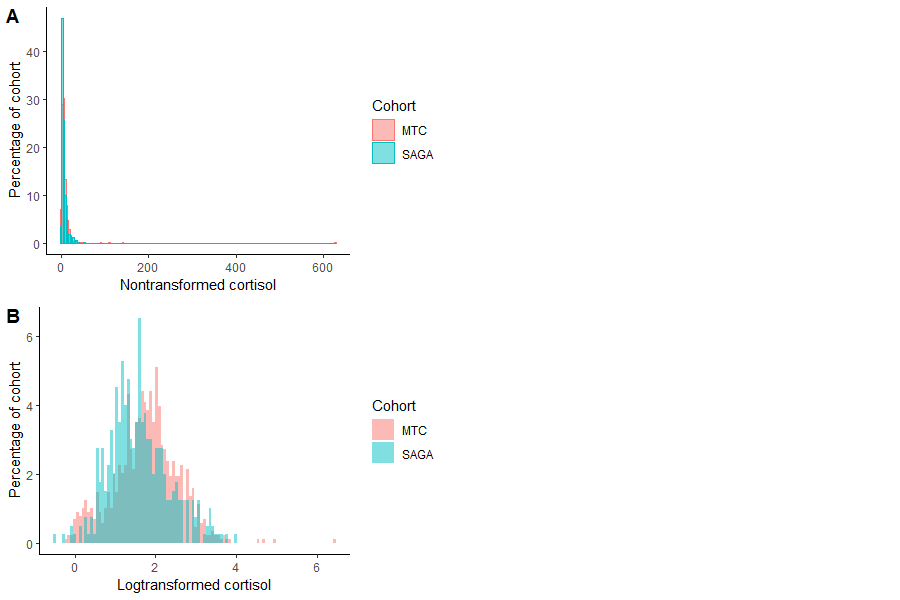

Supplement: S1 Fig — (TIFF) [file pgph.0000571.s001.tiff]

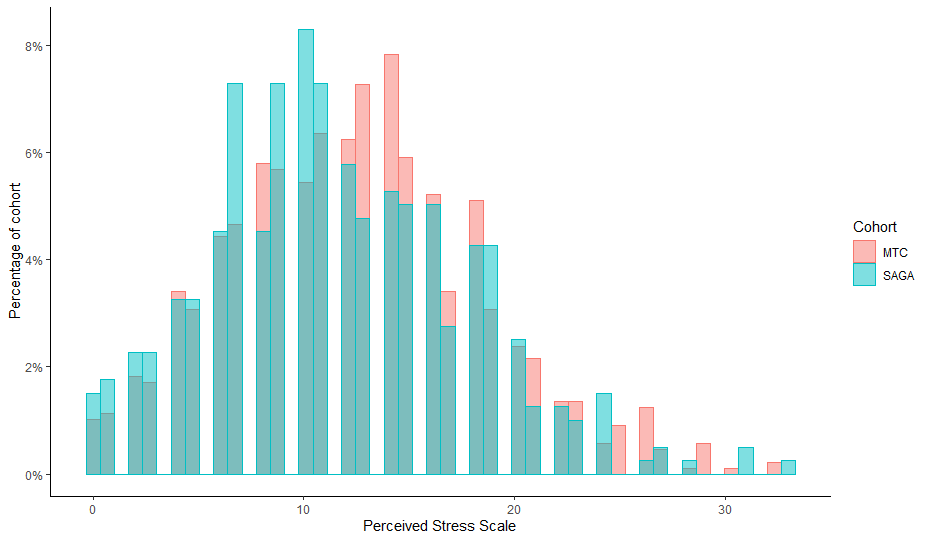

Supplement: S2 Fig — (TIFF) [file pgph.0000571.s002.tiff]

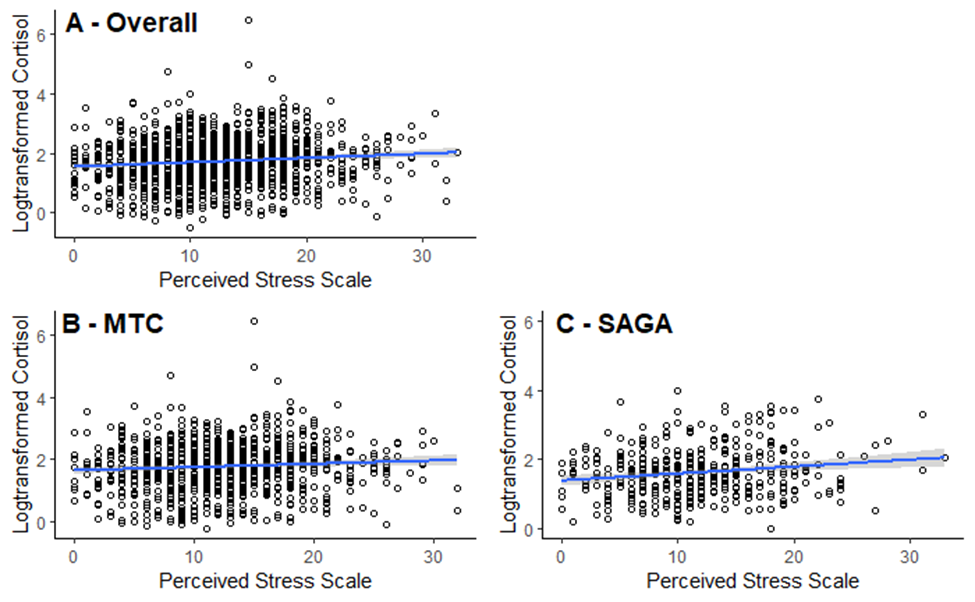

Supplement: S3 Fig — The figures are scatterplots with an overlying linear regression and 95% confidence intervals. The overall sample (Panel A) is age and cohort adjusted, with an 1.3% (95% CI 0.6, 2.1) increase in log-cortisol per unit increase of PSS (p value 0.001). The Mexican sample (Panel B) is age adjusted, with a 1.0% (95% CI 0.1, 1.9) increase in log-cortisol (p value 0.04). The Icelandic sample (Panel C) is age adjusted, with a 2.1% (95% CI 0.9, 3.4) increase in log-cortisol (p value 0.004). (TIF) [file pgph.0000571.s003.tif]

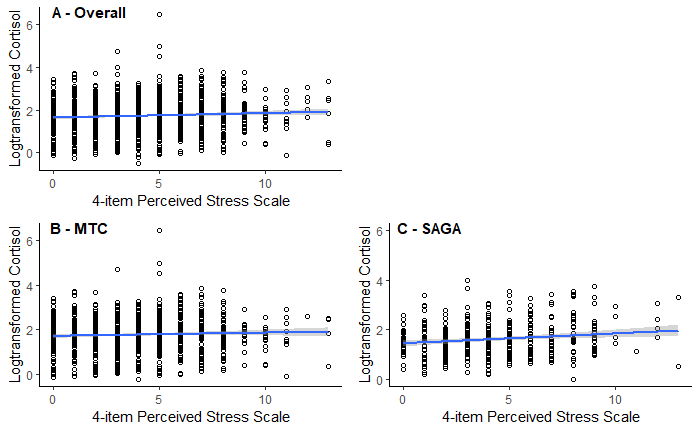

Supplement: S5 Fig — The figures are scatterplots with an overlying linear regression and 95% confidence intervals. The overall sample (Panel A) is age and cohort adjusted, with a 2.3% (95% CI 0.7, 4.0) increase in log-cortisol per unit increase of the 4-item PSS (p value 0.010). The Mexican sample (Panel B) is age adjusted, with an 1.4% (95% CI -0.6, 3.4) increase in log-cortisol (p value 0.212). The Icelandic sample (Panel C) is age adjusted, with a 3.9% (95% CI 1.1, 6.7) increase in log-cortisol (p value 0.008). (TIF) [file pgph.0000571.s005.tif]
