# Supplementary material for: Perceived stress and hair cortisol concentration in a study of Mexican and Icelandic women
Source: PLOS Glob Public Health. 2022 Aug 3;2(8):e0000571. doi: 10.1371/journal.pgph.0000571 (PMC10021558; doi:10.1371/journal.pgph.0000571)
Supplement: S1 Table — (DOCX) [file pgph.0000571.s007.docx]

**S1 Table. Description of variables used from the Mexican Teacher’s Cohort (MTC) (N= 881) and the Icelandic SAGA Cohort (N=398), their harmonization and missingness.**

| **Harmonized variable** | **Mexican Teacher’s Cohort** | | | **SAGA** | | | **Final**  **variable type** |
| --- | --- | --- | --- | --- | --- | --- | --- |
|  | **Collection method** | **Variable type** | **Missing (%)** | **Collection method** | **Variable type** | **Missing (%)** |  |
| Age | 2016-2017 Clinical visits | Continuous:  Calculated from birth year | 0 (0%) | 2014 online questionnaire | Continuous:  Calculated from birth year | 0 (0%) | Age (continuous, years) |
| Marital status | 2008 written questionnaire | Categorical:  Married*  Co-habitating*  Divorced†  Single †  Widowed† | 0 (0%) | 2014 online questionnaire | Categorical:  Married*  Co-habitating*  Relationship without cohabitation†  Divorced†  Single†  Widowed† | 2 (1%) | Categorical:  Partnered*  Single† |
| Education level | 2008 written questionnaire | Categorical:  Highest level completed:  High school  Bachelor  Masters  PhD | 0 (0%) | 2014 online questionnaire | Categorical:  Highest level completed:  Compulsory*  Upper secondary*  Vocational*  University   - B.A/B.S/B.Ed* - Diploma* - M.A/M.S/M.Ed† - PhD† | 2 (1%) | Categorical:  Graduate degree not completed*  Graduate degree completed† |
| Employment | 2017 Administrative data base | Categorical:  Employed*  Retired† | 0 (0%) | 2014 online questionnaire | Categorical:  Employed*  Retired†  Unemployed††  Disability††  Student†† | 8 (2%) | Categorical:  Employed*  Retired†  Other†† |
| Smoking status | 2016-2017 Clinical visits | Categorical:  Never smoker*  Former smoker†  Current smoker†† | 0 (0%) | 2014 online questionnaire | Categorical:  Never smoker*  Former smoker†  Current smoker†† | 1 (0%) | Categorical:  Never smoker*  Former smoker†  Current smoker†† |
| Alcohol consumption | 2008 written questionnaire | Categorical:  Never  ≤1 drink/ month  2-3 drinks/month  1 drink/week  2-4 drinks/week  5-6 drinks/week  1 drink/day  2-3 drink/day  4-5 drinks/day  ≥ 6 drinks/day  Median for each category calculated | 95 (10.8%) | 2014 online questionnaire | Categorical:  Drinks per month:  0  1-4  5-9  10-14  15-19  20+  Median for each category calculated | 5 (1%) | Continuous:  Drinks per month |
| BMI | 2016-2017 Clinical visit | Continuous:  height (m) and weight (kg) measured by trained study personnel. | 5 (0.6%) | 2014 clinical visit | Continuous:  height (m) and weight (kg) measured by registered nurses | 2 (1%) | Categorical:  Normal weight (BMI <25 kg/m^2^) Overweight (BMI 25 kg/m^2^ to 30 kg/m^2^) Obese (BMI >30 kg/m^2^) |

Variables that are grouped together with *, †, or †† were merged together for the final dataset.
