# Supplementary material for: Perceived stress and hair cortisol concentration in a study of Mexican and Icelandic women
Source: PLOS Glob Public Health. 2022 Aug 3;2(8):e0000571. doi: 10.1371/journal.pgph.0000571 (PMC10021558; doi:10.1371/journal.pgph.0000571)
Supplement: S2 Table — (DOCX) [file pgph.0000571.s008.docx]

**S2 Table. Characteristics of study participants (N= 1,279) , combining data from the Mexican Teacher’s Cohort (MTC) and the Icelandic SAGA Cohort, by quintiles of the Perceived Stress Scale.**

|  | **1^st^ Quintile** | **2^nd^ Quintile** | **3^rd^ Quintile** | **4^th^ Quintile** | **5^th^ Quintile** |
| --- | --- | --- | --- | --- | --- |
| **Hair Cortisol Concentration** |  |  |  |  |  |
| Median cortisol (pg/mg) | 5.0 | 5.1 | 5.7 | 5.7 | 6.3 |
| 25^th^ -75^th^ percentile | 3.1 – 7.7 | 2.8 – 8.3 | 3.5 – 8.9 | 3.7 – 9.4 | 3.6 – 10.2 |
| Mean log-cortisol (SD)^a^ | 1.6 (0.7) | 1.6 (0.8) | 1.7 (0.7) | 1.8 (0.8) | 1.8 (0.8) |
| **Mean age (SD)^a^** | 52.8 (7.8) | 52.4 (6.4) | 51.1 (8.0) | 50.1 (8.1) | 49.8 (7.9) |
| **Age in years (%)** |  |  |  |  |  |
| 20 – 39 | 10 (3.9) | 5 (2.0) | 16 (6.2) | 20 (7.8) | 21 (8.2) |
| 40 – 49 | 70 (27.3) | 71 (27.7) | 84 (32.8) | 81 (31.6) | 80 (31.4) |
| 50 – 59 | 127 (49.6) | 149 (58.2) | 118 (46.1) | 133 (52.0) | 137 (53.7) |
| 60 – 70 | 49 (19.1) | 31 (12.1) | 38 (14.8) | 22 (8.6) | 17 (6.7) |
| **Graduate Degree (%)** | 59 (23.0) | 53 (20.7) | 59 (23.0) | 49 (19.1) | 41 (16.1) |
| **Occupation (%)** |  |  |  |  |  |
| Employed | 229 (89.5) | 234 (91.4) | 225 (87.9) | 232 (90.6) | 228 (89.4) |
| Retired | 13 (5.1) | 12 (4.7) | 21 (8.2) | 7 (2.7) | 8 (3.1) |
| Other^b^ | 14 (5.5) | 10 (3.9) | 10 (3.9) | 17 (6.6) | 19 (7.5) |
| **Marriage/Cohabitation (%)** | 188 (73.2) | 184 (71.9) | 184 (71.9) | 183 (71.4) | 172 (67.4) |
| **BMI^c^ (SD)^a^** | 28.4 (5.6) | 29.2 (6.1) | 29.0 (6.0) | 28.7 (5.6) | 29.1 (5.6) |
| **BMI^c^ category (%)** |  |  |  |  |  |
| Normal weight | 72 (28.1) | 59 (23.0) | 71 (27.7) | 66 (25.8) | 60 (23.5) |
| Overweight | 99(38.7) | 112 (43.8) | 97 (37.9) | 100 (39.1) | 102 (40.0) |
| Obese | 85 (33.2) | 85 (33.2) | 88 (34.4) | 90 (35.2) | 93 (36.5) |
| **Smoking (%)** |  |  |  |  |  |
| Never | 160 (62.5) | 156 (60.9) | 160 (62.5) | 148 (57.8) | 139 (54.5) |
| Former | 70 (27.3) | 70 (27.3) | 68 (26.6) | 77 (30.1) | 66 (25.9) |
| Current | 26 (10.2) | 30 (11.7) | 28 (10.9) | 31 (12.1) | 50 (19.6) |
| **Alcohol consumption (drinks/month) (SD)^a^** | 3.5 (5.6) | 3.4 (5.6) | 3.3 (4.6) | 2.4 (4.0) | 3.3 (4.8) |
| **Mean PSS^d^ score (SD)^a^** | 4.3 (2.1) | 8.7 (1.0) | 12.0 (0.9) | 15.2 (1.0) | 20.9 (3.4) |
| **Mean PSS^d^-4 score (SD)^a^** | 1.1 (1.1) | 2.6 (1.3) | 3.7 (1.4) | 5.1 (1.4) | 7.8 (1.9) |
| **Cohort** |  |  |  |  |  |
| MTC | 152 (59.4) | 176 (68.8) | 183 (71.5) | 184 (71.9) | 184 (72.2) |
| SAGA | 104 (40.6) | 80 (31.2) | 73 (28.5) | 72 (28.1) | 71 (27.8) |

^a^ Standard deviation

^b^ Unemployed, disability, homemaker, student or sick leave

^c^ Body Mass Index

^d^ Perceived Stress Scale
